# Multiport Programmable Silicon Photonics Using Low-Loss Phase Change Material Sb_2_Se_3_

**DOI:** 10.1021/acs.nanolett.5c05838

**Published:** 2026-04-20

**Authors:** Thomas W. Radford, Latif Rozaqi, Idris A. Ajia, Priya Deoli, Xingzhao Yan, Mehdi Banakar, David J. Thomson, Ioannis Zeimpekis, Alberto Politi, Otto L. Muskens

**Affiliations:** † School of Physics and Astronomy, 7423 University of Southampton, Southampton, SO17 1BJ, United Kingdom; ‡ Optoelectronics Research Centre, University of Southampton, Southampton, SO17 1BJ, United Kingdom; ¶ School of Electronics and Computer Science, University of Southampton, Southampton, SO17 1BJ, United Kingdom

**Keywords:** Programmable photonics, Phase change materials, Sb_2_Se_3_, transmission matrix, photonic processor

## Abstract

Achieving an optimal platform to enable compact, efficient, and scalable reconfigurability is critical for next-generation technologies for large-scale photonic processors. Optical phase-change materials (PCMs) offer a compelling solution, and in particular Sb_2_Se_3_ stands out for its ultra-low-loss characteristics. Here, we present an experimental platform capable of encoding multiport operations onto the transmission matrix of a compact multimode interferometer architecture on standard 220 nm silicon photonics. The multiport devices are clad with a thin film of Sb_2_Se_3_, and direct laser writing induces local perturbations to the refractive index. A range of multiport geometries from 2 × 2 up to 5 × 5 couplers are demonstrated, achieving simultaneous control of up to 25 matrix elements with programming accuracy of 90% relative to simulated patterns and consistent optical performance across the C-band. Our work establishes a pathway toward the development of large-scale reconfigurable multiport devices on areas several orders of magnitude smaller than interferometer meshes.

## Graphical abstract

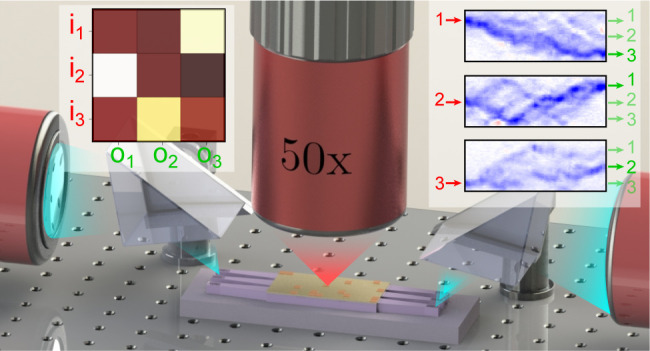

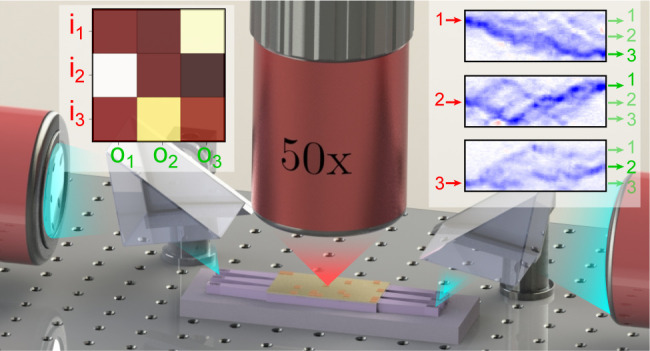

The development of a next generation of reconfigurable, large-scale photonic architectures signifies a critical milestone toward the realization of photonic matrix-vector multiplication accelerators,^1,2^ convolutional operators,^3^ optoelectronic neural networks,^4^ and microwave processors.^5^ The interface between nanophotonics and machine learning is currently receiving tremendous interest for the promise of achieving new platforms for optical information processing.^6–9^ Integrated photonic devices with addressable transmission matrices represent a foundational element in the advancement of analogue optical computing. Universal matrix decomposition has been well established using meshes of interferometers,^10–15^ yet such architectures typically occupy a large physical footprint even in modern, optimized configurations,^4,16^ which may ultimately require scale-up strategies beyond a single die.^17^

As an alternative to cascaded single-mode devices, the potential of exploiting complex wave interference in inverse designed multimode devices has raised substantial interest.^18–24^ Interference-based components such as multimode interferometers (MMIs) may be readily employed for matrix decomposition, leveraging controlled interference to direct optical signals to individual output ports, or for mixing states across several waveguides. These devices may be optimized during design,^23^ creating bespoke geometries for a given application. However, in the absence of reconfigurability, practical deployment remains limited, as most implementations are inherently static. Postfabrication control of silicon-based MMIs could be achieved through electrical heaters with limited freedom in designing the transfer function^25,26^ or all-optically using laser-induced perturbations.^27^

Next to electrically controlled active modulation,^8,14,28^ the development of nonvolatile reconfigurable platforms can offer longer-term adaptive functionalities, reduced energy footprint, and postfabrication diversification and trimming in high-volume manufacturing.^13,29–32^ In particular, low-loss optical phase-change materials (PCMs) offer a promising route to enable light modulation within an ultracompact footprint.^33–39^ Among the new generation of low-loss optical PCMs investigated in recent years,^37,38^ antimony triselenide (Sb_2_Se_3_) has become one of the main materials of choice for the development of reconfigurable silicon photonics owing to its favorable optical properties,^40,41^ with integration of these new PCMs into CMOS foundries ongoing.^42,43^ Reprogramming of Sb_2_Se_3_-based PCM devices has been demonstrated via electrical^36,44,45^ or optical actuation,^8,46,47^ and endurance of over one million write and reset cycles was shown using carefully selected parameters.^48,49^

In this work, we report the active reconfiguration of multimode, multiport devices and demonstrate the programming of permutation matrices with up to 25 elements. This new capability offers a critical step toward the development of new types of ultracompact devices for optical information processing. Our work uses a standard 220 nm silicon photonics platform combined with 30 nm thin films of Sb_2_Se_3_ to functionalize compact MMI geometries. This PCM thickness was chosen as a trade-off between insertion loss and modulation strength, allowing sufficient control per pixel at moderate pixel numbers per pattern to achieve matrix implementations.^50,51^ Using direct laser writing of digital patterns, we demonstrate that combinations of weak scattering perturbations can be employed to constitute a matrix operation within the continuously coupled, multiport device. Iteratively generated refractive perturbation patterns are used to achieve matrix operations within a greatly reduced footprint compared to other approaches.

In recent years, sophisticated inverse design methodologies have been developed, including topology optimization,^18,23,52,53^ nontopology blackbox optimization including direct binary search and genetic algorithms,^19,54–57^ and the application of machine learning and artificial intelligence techniques.^50,58,59^ For the purpose of this study a direct binary search approach was chosen to predict well-performing solutions for a small number of selected matrices using a 2.5D variational finite difference time domain (varFDTD) simulation engine. Details of the optimization are presented in Supporting Information Section S1. Shape similarity between the final optimized and target matrices is quantified using the average cosine similarity between output vectors. Cosine similarity is a dimensionless and scale invariant metric describing the mutual alignment of two normalized vectors through the cosine of their angle. Next to cosine similarity we consider the device throughput or insertion loss obtained from the total transmission over all output ports.

Devices were fabricated using the 220 nm UK CORNERSTONE silicon-on-insulator (SOI) platform^60^ using the same methods as in previous work.^40,46,51^ A schematic cross-section of the device stack is shown in the inset of Figure 1a. The SOI rib waveguides were etched 120 nm into the top silicon layer of the wafer. A 30 nm thick layer of Sb_2_Se_3_ was sputter coated onto the devices using an Sb_2_Se_3_ target (Testbourne) at an RF power of 35 W, 5.75 mTorr, and 25 sccm of Ar, before a lift-off process allowing selective deposition of PCM onto active regions of the devices. Final structures were cladded with a 80 nm thin ZnS:SiO_2_ (20%:80%) capping layer (20 nm directly after PCM deposition and 60 nm following lift-off) to prevent oxidation of the PCM and to enhance the switching performance of individual pixels. Prior to Sb_2_Se_3_ deposition, a short argon-ion etch was employed to remove any surface oxide. Layer thicknesses were measured using ellipsometry and found to be within 5 nm from target values. The multimode regions of the 2 × 2, 3 × 3, and 4 × 4 MMI devices under study have lateral dimensions of 6 × 40 μm^2^ and feature tapered waveguides at input and outputs, which aid to reduce losses during outcoupling. The 5 × 5 MMI is slightly wider to accommodate all the ports, with a dimension of 8 × 40 μm^2^.

**Figure 1:**
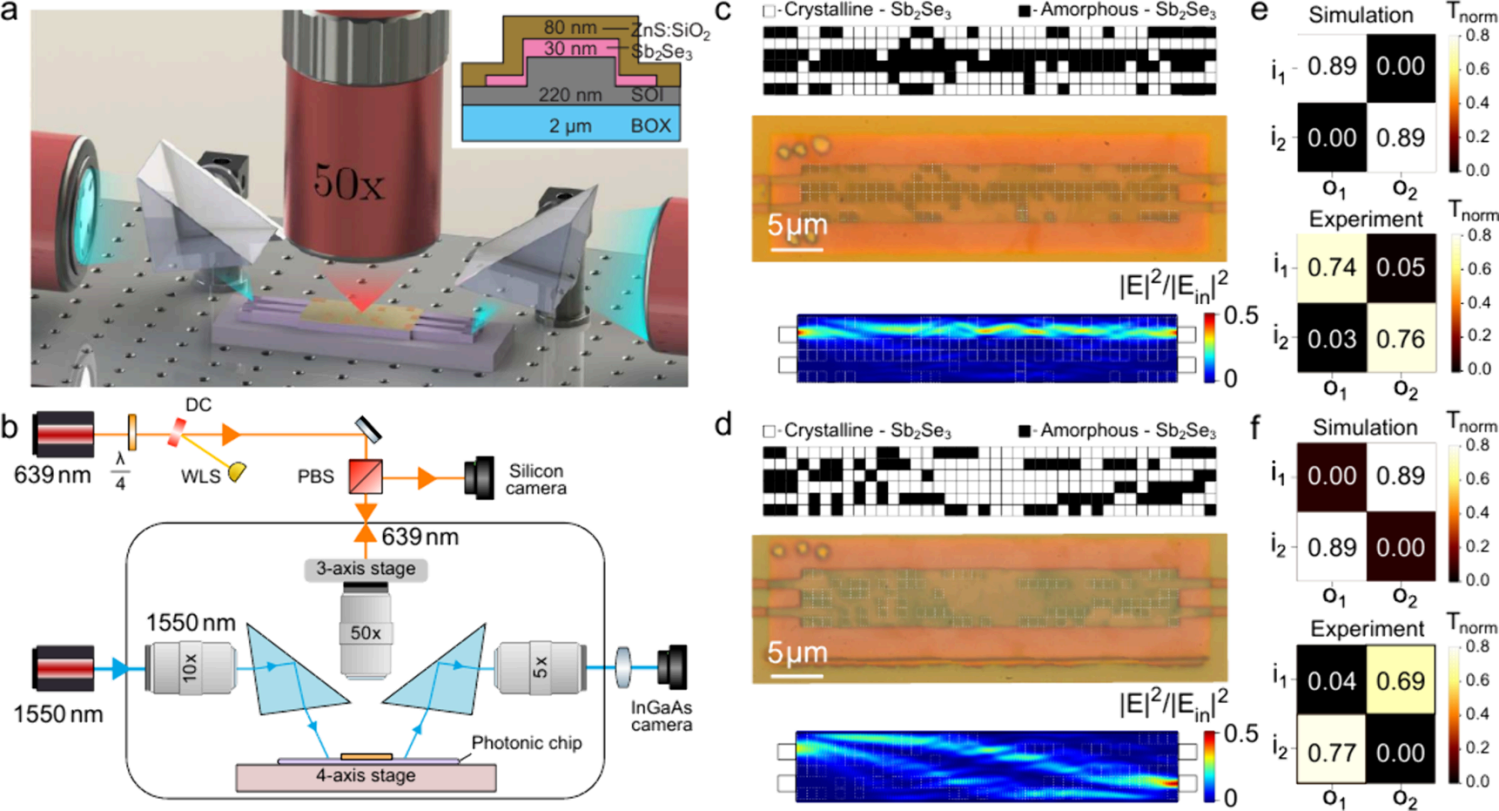
(a) 3D artistic rendering of the constructed contact-free prism coupler used for direct laser writing of PCM films as well as device characterization. (b) Detailed schematic of the experimental setup. (c, d) Experimental results for the bar (c) and cross (d) states of the 2 × 2 multiport switch with designed perturbation maps (top), photograph of MMI after switching (middle), and calculated near-field map (|*E*|^2^) for top input (bottom). (e, f) Simulated (top) and experimental (bottom) transmission matrices for the two states.

The PCM was amorphous as-deposited and was subsequently crystallized using a hot plate at 200 °C for 10 min. Subsequent optical switching of Sb_2_Se_3_ was achieved through direct laser writing using a current-modulated diode laser at 639 nm wavelength (Vortran Stradus), using pulses of 80 ns at 90–120 mW for amorphization and 200 ms at 45 mW for crystallization. More information about specific switching parameters can be found in the Supporting Information.

The experiment requires simultaneous access to multiple device ports. However, space is restricted by the 17 mm working distance of the microscope. Therefore, a customized setup was built using a pair of free-space noncontact prisms for coupling in and out of the devices. A 3D rendering outlining the complete experimental process is shown in Figure 1(a) with an experimental schematic provided in Figure 1(b) and more detail outlined in the Supporting Information Section S1.2. A fiber-coupled tunable laser source was focused through the input prism using a 10× microscope objective and coupled into a single input waveguide of the device. On the output side, multiple grating couplers were imaged simultaneously onto an InGaAs camera. Areas of interest corresponding to individual grating outputs were selected to quantify the relative intensity distribution across the output ports using postprocessing in Python. Experimental matrix elements were normalized to a straight waveguide reference.

As a first demonstration we investigated the switching of a 2 × 2 MMI splitter. Programmable two-port interferometers are essential building blocks for universal optic gates in mesh-based optical computing, and this relatively basic device geometry represents an important use case. In the unperturbed state (crystalline background), the device geometry was not optimized for any particular self-imaging condition and showed significant out of plane scattering of input light and insertion loss exceeding 7.5 dB. Programming of the device resulted in the concerted action of the perturbations guiding light toward the targeted output waveguides. The chosen matrices implement an identity transformation (bar state, Figure 1c,e) and its inverse (cross state, Figure 1d,f). The simulated near-field maps for the top input show that the designed pattern efficiently guides light to the corresponding output; similar simulation results are obtained for the other port (Supporting Information). In the simulation, a splitting ratio of 100%:0% was obtained for both states; experimentally we achieve values better than 93%:7% for all ports, or a port extinction of 11.2 dB. The total device transmission normalized to a straight waveguide is around 75%, indicating an insertion loss of 1.2 dB. Due to the spatial separation in the optical path for the identity transformation, light may be routed with low crosstalk by selecting a geometry that switches straight paths of PCM to the amorphous state, effectively forming low-index channels that guide light akin to waveguides.^61,62^

Provided a sufficient ability to achieve suitable design predictions, the technique should be able to program arbitrarily large devices with a comparable degree of accuracy. Larger multiport devices offer a broader range of potential applications, such as more powerful analogue simulation of complex systems.^23^ To test our experimental capability, we attempted programming of 3 × 3, 4 × 4, and 5 × 5 MMI geometries where we extend the number of degrees of freedom respectively to 9, 16, and 25 matrix elements. Each multiport coupler has many available permutation matrices; here we selected one target design for each device geometry, resulting in the designed pixel patterns shown in Figure 2a and simulated transmission matrix in Figure 2b. Increasing the number of matrix elements significantly increases the challenge of finding a single designed pattern able to implement the desired transmission target. Still, our simulations achieve agreement to within 92% of the ideal permutation matrix, as defined by the cosine similarity of each port, and optimized port transmissions exceeding 80% or 0.9 dB insertion loss, matching previous more in-depth modeling efforts for a similar device geometry for the 3 × 3 geometry.^50^ Experimental results for the patterned devices are also shown in Figure 2a and b. The difference matrix for each case is shown in Figure 2c, which represents the experimental transmission minus the simulated transmission. Port transmissions exceeding 70% are achieved for all ports where generally the peak intensity is reduced compared to the simulation (negative difference, blue in Figure 2c). At the same time, other nonoptimized ports show a nonzero background intensity above the simulated (positive difference, red in Figure 2c), which is attributed to imperfections in routing the signal to the output through the multiple interference of weakly scattered light paths. These positive backgrounds together are slightly higher than the negative peak loss, resulting in a small positive residual of around +0.15 when summed for each input, indicating that the total throughput of the experimental devices is slightly higher than the simulated ones. However, this additional transmission is largely contained in the nonoptimized ports.

**Figure 2:**
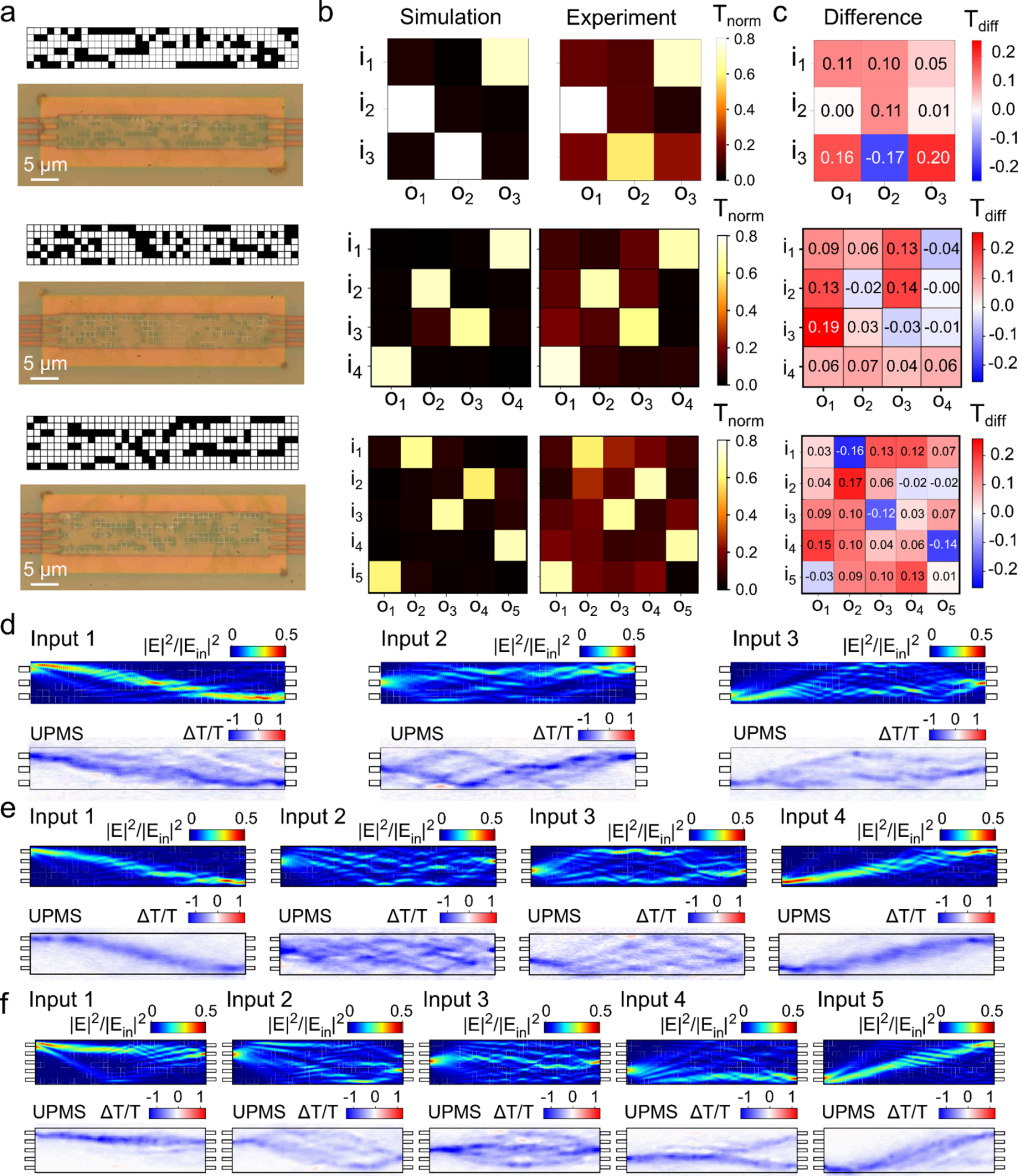
(a) Photographs of programmed MMI devices designed to implement 3 × 3, 4 × 4, and 5 × 5 permutation matrices. (b, c) Simulated and experimental transmission matrices (b) for the programmed devices normalized to a straight waveguide reference and their difference (c), demonstrating average programming errors of ∼12% and an average cosine similarity in excess of 0.9. (d–f) Simulated near-field intensity |*E*|^2^ maps and ultrafast photomodulation spectroscopy (UPMS, port-summed) measurements probing the local intensity distribution within patterned devices, for the three different devices of (a–c).

To complement our transmission matrix measurements and gain more insight into the flow patterns generated by the perturbed MMIs, we performed ultrafast photomodulation spectroscopy (UPMS) mapping. UPMS is an ultrafast pump–probe technique developed in our previous work^63,64^ which can be used to map the local intensity distribution in programmed devices.^50,51^ In UPMS an ultrafast laser is scanned over the device area and induces a local refractive index perturbation. The effect of this perturbation on the probe transmission is measured for each combination of input and output ports. Details of the technique are presented in the Supporting Information Section S3. Figure 2d–f show maps of the UPMS differential transmission response obtained by summing the individual signals from all output ports for each of the device inputs, for the 3 × 3 (d), 4 × 4 (e), and 5 × 5 (f) MMI geometries. These port-summed maps can be directly compared with the local intensity inside the device.^63^ Corresponding device simulations are presented in the same figures and provide a good qualitative agreement with the observed flow patterns and routing of the signal toward the different outputs. The UPMS maps also reveal some of the parasitic paths routing light toward undesired ports. Overall the experimental maps confirm the operating principle of the programmable MMIs whereby complex flows of light are being produced by multiple weak scattering events in the digital perturbation pattern.

In Figure 3, we demonstrate the spectral stability of a typical patterned device. Transmission matrices were recorded at wavelengths across the telecommunications C-band range from 1530 to 1570 nm. Recorded transmission values were normalized relative to a straight waveguide at the same test wavelength to compensate for the spectral response of the measurement system and grating couplers. Figure 3a,b plots a selection of matrices from across this range together with the cosine similarity between the simulated device performance at 1550 nm and the experimentally recorded transmission matrix at 1 nm increments. Targeted ports (i1:o4, i2:o2, i3:o3, and i4:o1) remain the dominant source of transmission at all studied wavelengths with relative matrix element deviations of around 11%. The programmed devices generally show low insertion losses and offer broadband compatibility across all C-band wavelengths with minimal deviations in the transmission matrix shape from those recorded at 1550 nm, the wavelength for which the patterns were designed. Simulations presented in Supporting Information Figure S4 show a consistent theoretical transmission of around 70% for both the total and per-port values across all test wavelengths. This spectral bandwidth enhances the applicability of the platform for systems requiring multiwavelength operation or flexible source integration and can likely be improved upon with the use of more advanced inverse design considering also the spectral bandwidth.

**Figure 3:**
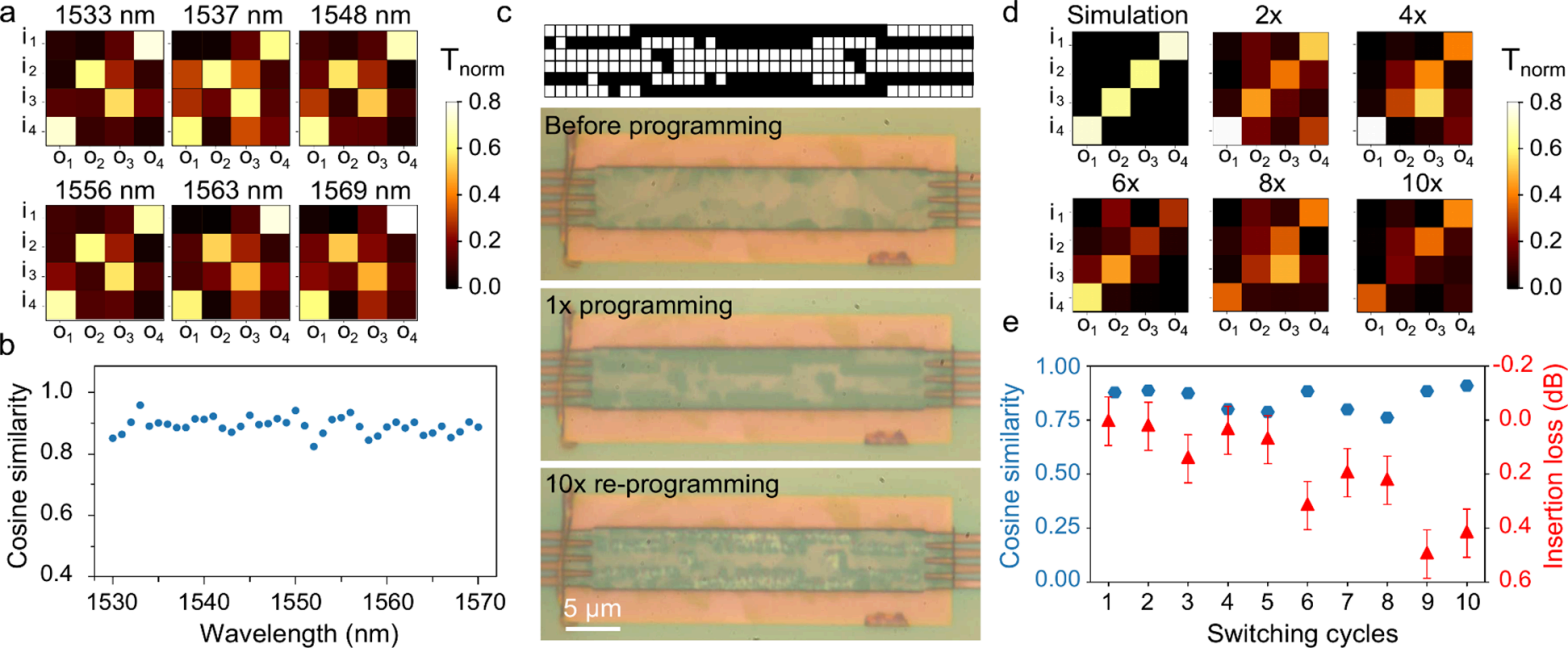
(a, b) Spectral response across C-band wavelengths of a patterned 4 × 4 MMI as outlined in Figure 2, with experimental transmission matrices at five selected wavelengths (a) and cosine similarity of experimental matrices at 1 nm wavelength steps with simulated design matrix at 1550 nm wavelength. (c–e) Programming endurance study for 4 × 4 MMI using direct laser writing for both set and reset transitions. (c) Simulated pixel pattern design and photographs of the device before programming, after the first programming step, and after 10× reprogramming cycles including a recrystallization. (d) Simulated and experimental transmission matrices for the initial design and for between 1× and 10× reprogramming cycles. (c) Port-averaged cosine similarity and excess insertion loss versus number of reprogramming cycles.

A further important test is to ensure that a single device geometry is able to withstand multiple switching cycles. To this end Figure 3c–e present results for a 4 × 4 MMI, programmed using a pixel pattern designed to implement an inverse identity transformation. The device is optically programmed and subsequently deprogrammed, allowing study of the transmission characteristics throughout this process. The cosine similarity in Figure 3e is taken between the simulated matrix and the recorded experimental matrices for each reprogramming cycle. Through repeated switching small defects develop in the capping layer of the material, which results in increased insertion losses of up to around 0.4 dB after 10 cycles. Capping layer defects can be observed as lighter spots remaining after thermal annealing of the sample following the 10 programming runs seen in the microscope image of Figure 3c, bottom panel. Switching-induced nonuniformity in the capping layer could be caused by the mechanical expansion and stresses in the film.^48^ While our work indicates that repeated switching is possible, further work will need to be directed toward optimization of pulse parameters and mechanical stability of the stack.

Scalability of the approach toward larger multiport systems depends on availability of inverse-design algorithms employed and from the accuracy with which patterns can be written into the PCM film. As the device size increases, two compounding optimization challenges emerge: the number of matrix elements requiring optimization grows quadratically with port count, and the combinatorial pixel space expands exponentially with increasing device area, presenting a clear target for future research. In our studies FDTD simulations were separately validated using experimental UPMS mapping, demonstrating good agreement with our simulation models. Other works have demonstrated scaling of computational design up to 10 × 10 ports.^23,65^ These available studies and our own work jointly provide evidence of the suitability of the multimode platform for a range of complex programming challenges.

Average programming errors (the difference between simulated and recorded performance) across all presented matrices remain around 10% on average; however, experimental optimization of nontargeted ports is noticeably poorer than in simulation. This suggests that a significant source of experimental error originates from small deviations between the simulated and the programmed device. In particular, variations in spot size, shape, and pixel placement are of key importance, as even minor changes to the multimode regions of MMI geometries can dramatically alter output coupling due to the reliance on precise positioning of self-imaging points. Additional simulation studies are presented in the Supporting Information, addressing the critical role of alignment accuracy of the pattern onto the MMI region, as well as the role of variations in the PCM thickness known to result in different switching contrast Δ*n*.^51^ It is seen that transverse alignment within the MMI region is particularly critical for performance. As device dimensions grow, the importance of accurate programming further increases, as small inaccuracies compound across the larger number of programming sites required to tune the output of larger devices. Future work will therefore prioritize improvements in both the resolving power of the microscope objective and the lateral resolution of the programming stage to more faithfully reproduce simulated device geometries.

Within the landscape of programmable photonic processors, multimode devices are of interest for their ability to offer a small footprint compared to implementations using coupled single-mode waveguides. Figure 4 places this in perspective by comparing the footprint of the MMI-based multiport devices with state-of-the-art photonic processors. Here, we have made use of the survey data provided by Pérez-López and Torrijos-Morán^1^ comprising a recent overview of the field. Data are presented in similar form separating different technological approaches including interferometer meshes, resonators, phase arrays, and other technologies such as MEMS and bimodal waveguides. Figure 4 plots the total device footprint against the total number of optical ports (inputs and outputs), where only devices with more than one optical port are presented for clarity. Multimode interferometer (MMI) approaches stand out for their strongly reduced surface area by several orders of magnitude. In general, for any multiport operator, the device width *W* is assumed to be proportional to the number of diffraction-limited ports that can be fitted; *W* = *Nλ*_0_ where *N* denotes the number of independent input ports and λ_0_ is the vacuum wavelength. However, the scaling of device length *L* with the number of input ports *N* depends critically on the method used to couple the channels, either through cascaded two-port operations or through multimode interference. Recent work by Onodera et al.^66^ discussed this scaling for weakly perturbed multimode interferometers and derived an expression for the total area (*L* × *W*) proportional to $N^{3/2}\lambda_{0}^{2}/\Delta n$ , where Δ*n* is the difference in effective mode refractive index that can be achieved using the given material platform. In comparison, traditional cascaded two-port interferometer meshes have a footprint *L* × *W* that scales roughly quadratically with the number of ports as $N^{2}\lambda_{0}^{2}/\Delta n$ . The PCM-based programmable MMIs have a large difference in effective mode index between states of Δ*n*_PCM_ = 0.1,^51^ resulting in the scaling trend for the MMI-type devices as shown by the brown dashed line in Figure 4. In comparison, the typical refractive index modulation in electro-optical or thermo-optical systems is much smaller at Δ*n*_EO_ ≃ 3 × 10^–3^,^67^ resulting in a corresponding scaling for mesh-based approaches as shown by the black dashed line in Figure 4.

**Figure 4:**
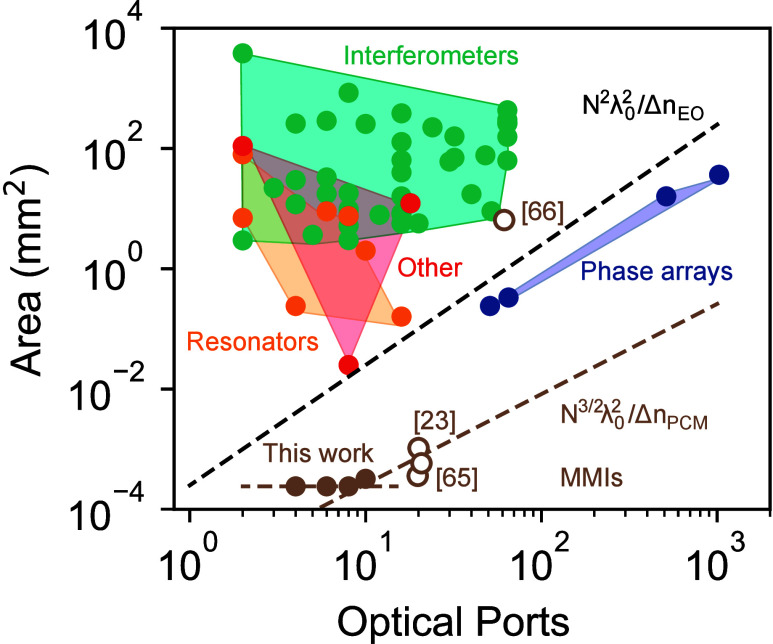
Device area versus total number of optical ports plotted for selected devices with >1 ports from the survey of Pérez-López and Torrijos-Morán^1^ classified into interferometers (green), resonators (orange), phase arrays (blue), and other (red). Additionally results are presented for experimental MMIs in this work, as well as from refs 23, 65, and 66. Dashed lines represent theoretical scaling behavior^66^ for cascaded interferometer meshes and multimode interferometers for the typical effective mode index difference between states for electro-optics^67^ (Δ*n*_EO_ = 3 × 10^–3^) and low-loss PCMs^51^ (Δ*n*_PCM_ = 0.1) assuming N equal to half of the total optical ports.

For comparison we have also included in the figure the recent result on lithium niobate programmable MMIs with up to 59 optical ports by Onodera et al.^66^ Their demonstrated device offers similar advantages of multimode interference, but exploiting photorefractive effects at much lower refractive index changes of around 10^–3^, resulting in a device footprint of around 9 × 1 mm^2^. A data point for the simulated 10 × 10 port silicon-based patterned MMI from Nikkhah et al.^23^ and two data points for the devices from Sved et al.^65^ are also shown. Their conceptual devices constitute etched holes in a silicon waveguide and are not fully programmable as in our approach, but their designs could be readily adapted to PCM-based technology with similar index contrast and hence following the same scaling (dashed brown line).

In conjunction with the aforementioned improvements in spatial selectivity of programming pixels, the programmable Sb_2_Se_3_-silicon integrated devices could enable compact and reconfigurable implementations of arbitrary unitary transformations, with potential future applications in programmable photonic circuits for optical logic, analog computing, and neural networks. Once patterned, the device operates without the need for active control or regulation, thereby enabling a variety of emerging optical technologies. Reconfigurable devices utilizing Sb_2_Se_3_ are scalable, owing to the minimal footprint and resolution of perturbation sites, limited only by the employed fabrication process and experimental optics chosen. PCM integration of multiport interferometers enables complex matrix decomposition within a micrometer-scale, continuously coupled architecture, demonstrating a dramatic reduction compared to conventional approaches that rely on large two-port interferometric meshes which often require active stabilization. While our current work focuses on permutation operators of intensity, future studies should address the potential for the programming of complex unitary operators. This will, however, require considerably more sophisticated methods for phase measurement at the output ports going beyond our current capabilities.

In conclusion, we have demonstrated the repeated nonvolatile programming capabilities of integrated photonic devices clad with thin films of antimony triselenide. Using direct laser writing, we were able to experimentally map a range of target matrix operations onto a number of multiport MMI devices, highlighting both the versatility and scalability of this approach, but also pointing out some limitations in the current implementation related to imperfections in the coupling and leakage to other ports, as well as in the repeatability studies. The patterned devices exhibit increased coupling efficiency with respect to plain MMIs of the same geometry, while maintaining modest insertion losses even for larger 5 × 5 port geometries. Resulting devices allow full control over the amplitude of all available input–output combinations and achieve an average programming accuracy of 90.7% relative to simulated device models, underscoring their suitability for a broad range of experimental systems. The proposed platform holds strong potential for applications in optical logic, photonic processors, AI, and quantum simulation, where compact, reconfigurable, and power-efficient components are essential.

## Supplementary Material



## Data Availability

Supporting data used in this work are openly available from the University of Southampton repository at doi.org/10.5258/SOTON/D3764.

## Notes

Experimental method description with a schematic of setups for optical switching and UPMS, optical switching parameters, additional experimental results for optical programming, additional simulation results and sensitivity to pattern alignment and PCM film thickness (PDF)
