# Supplementary material for: Multiport Programmable Silicon Photonics Using Low-Loss Phase Change Material Sb2Se3
Source: Nano Lett. 2026 Apr 20;26(16):5370–8. doi: 10.1021/acs.nanolett.5c05838 (PMC13133916; doi:10.1021/acs.nanolett.5c05838)
Supplement: Supplementary file 1 [file nl5c05838_si_001.pdf]

Supporting Information: Multi-port programmable silicon photonics using low-loss phase change material Sb_2Se_3.

Thomas W. Radford,[†,§] Latif Rozaqi,[†,§] Idris A Ajia,[†] Priya Deoli,[†] Xingzhao Yan,[‡] Mehdi Banakar,[‡] David J Thomson,[‡] Ioannis Zeimpekis,[¶,‡] Alberto Politi,[†] and Otto L. Muskens[*,†]

†*School of Physics and Astronomy, University of Southampton, Southampton, SO17 1BJ, United Kingdom*

‡*Optoelectronics Research Centre, University of Southampton, Southampton, SO17 1BJ, United Kingdom*

¶*School of Electronics and Computer Science, University of Southampton, Southampton, SO17 1BJ, United Kingdom*

§*These authors contributed equally.*

E-mail: O.muskens@soton.ac.uk

1 Methods

The following sub-sections outline the methods and techniques used for the simulation, fabrication, and testing of the programmed MMI devices presented in the main body of the text.

1.1 Numerical simulations

Numerical simulation is a crucial tool required to determine a suitable pixel pattern to implement a given transmission matrix. The unperturbed MMI geometry is based around a standard 220 nm SOI fabrication process. Figure S1 shows a schematic of the fabricated geometry for a 3x3 MMI, as well as a cross section of the device to highlight the etch and deposition steps. The width of 6 μm of the multi-mode region is specific to the 3-port device. For devices with a larger number of input/output waveguides a greater width is required in order to accommodate the waveguide tapers which are 20μm long and a final width of 1μm. These are added to reduce optical losses when coupling light into and out of the device.

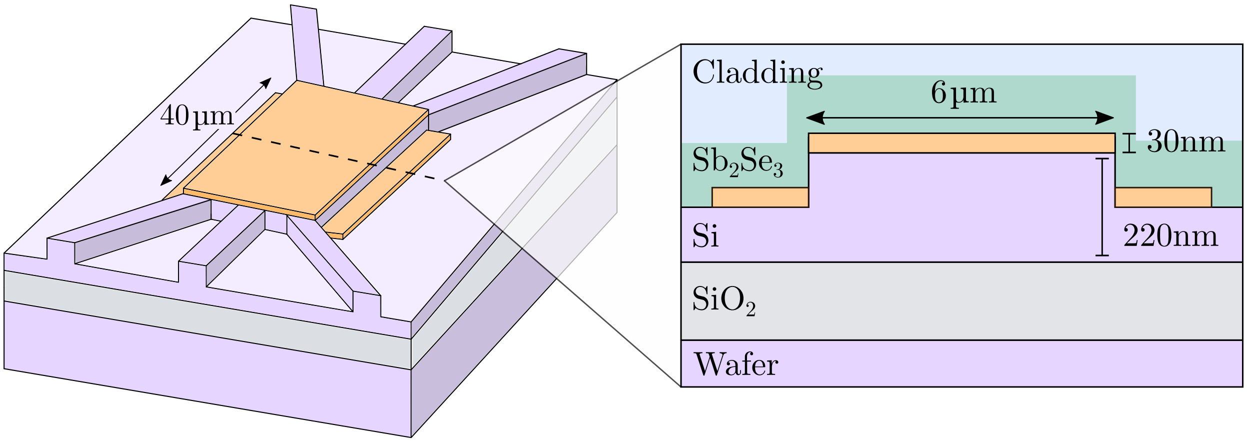

Figure S1: Schematic of the MMI geometry used in this paper. A 30 nm Sb_2Se_3 layer is deposited on top of the MMI region of the device, designed around a standard 220 nm SOI rib waveguide fabrication process. The presented 6 μm wide multimode region allows a maximum of 4 input/output ports, after which this is increased to 8 μm.

Our optimization process begins with the unperturbed MMI device as shown in Figure S1, the Sb_2Se_3 film is initialized in its crystalline state and divided into a predefined grid of pixels. A target transmission matrix is defined within Lumerical, and a pixel selected at random. The state of this pixel is switched to its amorphous phase (dark pixels in the presented figures), and the modified device simulated. A cost function is used to evaluate the similarity between the transmission matrix of the device before and after the selected pixel is

switched. If the new configuration results in improved agreement, the change is retained and another pixel is selected, if the agreement is reduced, the pixel is reverted to its prior state. This process is repeated for N pixels, changing the state between amorphous or crystalline where appropriate in pursuit of a global minimum of the cost function. After N iterations, the final pixel pattern is saved, allowing future programming via a python interface written for this work.

1.2 Direct-write experiment

All experimental measurements for transmission matrices presented in this work are recorded using the same contact-free prism coupler set up. The individual components used are discussed in the main text body, Figure S2 presents a clearer experimental set-up schematic, including a sample image taken from each of the available visible and infrared camera positions.

Two right angle glass prisms are positioned above the chip at a slight angle to allow optimal coupling using the waveguide gratings which are optimized for 10°incident light. A flip mirror is used to couple a broadband halogen IR light source through the input 10× microscope objective, allowing positioning of the input laser spot over the waveguide grating using a 1550 nm InGaAs camera at position (i).

After coarse alignment of the chip, the IR camera can remain in one of two locations using magnetic optical mounts. At location (i) a 4f pinhole system may be installed using a further magnetic mount. This allows measurement for the transmission of devices in which input and output waveguide gratings are on the same side of the device. Location (iii) instead allows the measurement of MMIs which have input and output gratings in opposing directions. Using this arrangement, the transmission of multiple output ports at once can be investigated by imaging up to 7 output gratings simultaneously (when using the 5× microscope objective shown for these results).

A visible camera is separately coupled into the top-down 50× microscope objective at lo-

cation (ii) using a polarizing beam splitter. This allows real time positioning and monitoring of the writing laser pulse upon the PCM film.

Recorded images from the InGaAs camera are converted into relative intensity measurements using post processing image analysis as shown in Figure SS3. By taking a linesweep across the image a 2D plot can be reconstructed, from which the integrated peaks are recorded. Following this normalization relative to the a neighboring straight waveguide device is carried out. This normalization allows isolation of recorded results from the spectral response of the grating couplers used to inject light onto the chip, and allows comparison between different devices and samples without concern of fabrication or deposition discrepancies altering results. It also allows calculation of relative insertion losses for the MMI itself before and after programming.

2 Optical Switching Parameters

Focused laser pulses of nanosecond time duration enable high-resolution, spatially selective programming through local amorphization of the PCM with individual pixel size of around 0.7 µm.[1] Experimentally, programming is carried out using high speed pulses of above-bandgap laser light, focused through a 50×, 0.42 NA long-working distance microscope objective (Mitutoyo Plan APO NIR), positioned above the sample as discussed above. An electronic pulse generator (BK precision 4063b) is used to control the pulse duration and optical power of the laser through a combination of digital and analogue voltage outputs. The objective was mounted on a three-axis stage assembly (Newport), which provided accurate positioning of the laser spot across the PCM region to program pixelated patterns of perturbations onto the PCM film.

Pulse parameters, for the digitally gated 639 nm are calibrated at the begining of each measurement using a test device neighboring the MMI of interest. Pulse energy is gradually

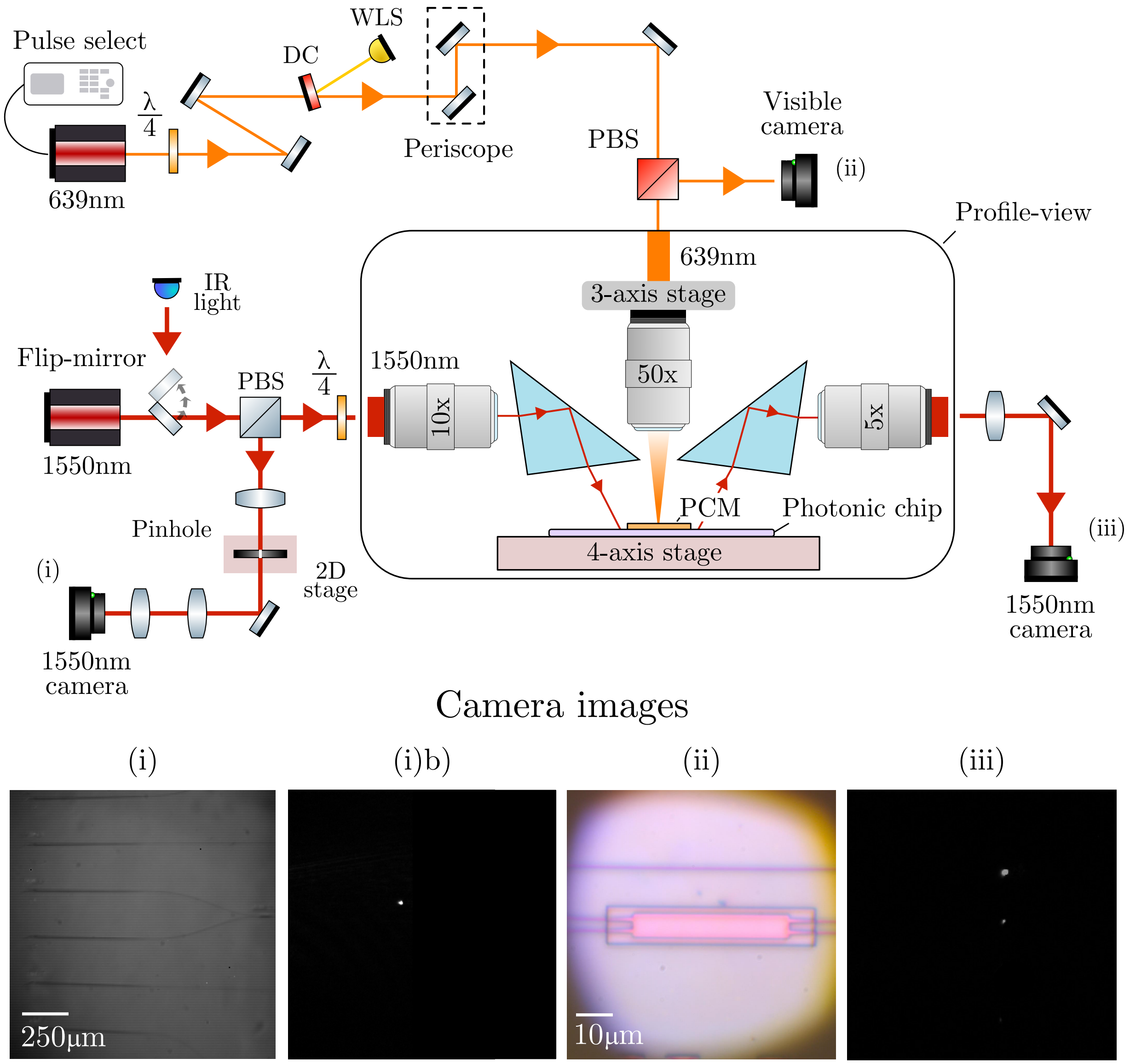

Figure S2: Experimental set up used for programming and characterisation of PCM clad photonic devices. Representative images are presented to show what signal may be measured for each position labelled in the schematic. Sub figure (i) and (i)b) represent the image without, and with the pinhole fitted respectively which is affixed using a removable magnetic mount.

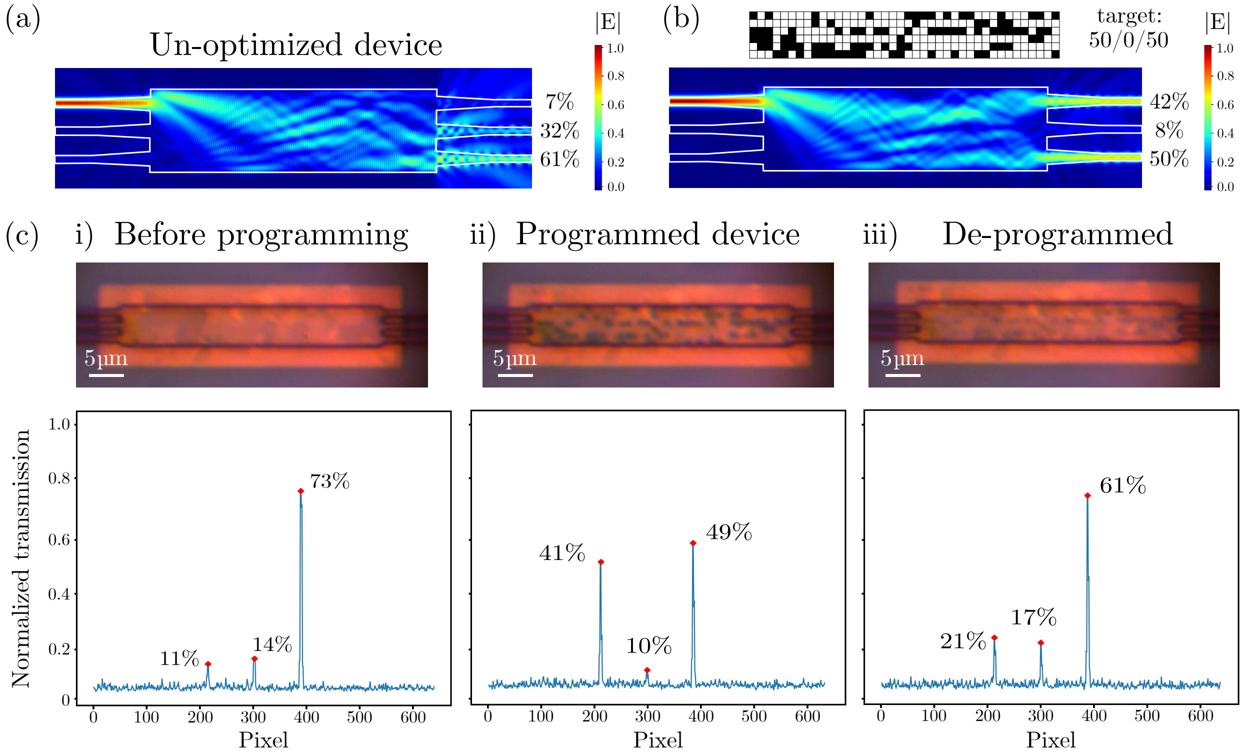

Figure S3: Image analysis for a patterned 3×3 MMI device. The pattern is selected to achieve 50/50 splitting between the top and bottom ports as shown in the simulated FDTD near field maps (b). Device performance is not completely reversible, likely due to small regions of damage caused by excessive laser power which can be seen in the top-down microscope images (c) after de-programming.

increased until a visible change in reflectivity of the spot is observed, at which point a ”reset” pulse is sent to ensure reversible programmability is achieved. Amorphization pulses were 80 ns long and varied between 90-120 mW nominal power from the laser source depending on the individual sample used. Crystallization pulses are consistently 200 ms in duration and delivered at 45 mW of laser power due to the reduced risk of damage to the cladding material from rapid changes in surface morphology. At times, variation in the thermal conductivity across the MMI can cause issues with switching (eg -edge pixels which have decreased material surrounding them to aid thermal transfer), in such cases pulse parameters may need to be adjusted to ensure the pixel pattern is reproduced faithfully to the design.

3 Ultrafast Photomodulation Spectroscopy

For UPMS measurements, the devices were transferred to a different fibre-coupled setup and individual port combinations were measured in a pump and probe configuration using an ultrafast pulsed laser at 1560 nm wavelength (Menlo C-Fibre) as the probe and a frequency quadrupled 390 nm UV laser as the pump. The pump was modulated at 5 MHz using an acousto-optic modulator (AA Opto-Electronic) and focused onto the surface of the MMIs by a 50× objective with a NA of 0.55 (Mitutoyo). A Thorlabs dual-axis galvo scanner with dielectric mirrors was used to scan the pump signal across the active area of the MMI. The change in probe signal due to the perturbation induced by the pump laser was recorded using a lock-in amplifier (Zurich Instruments).

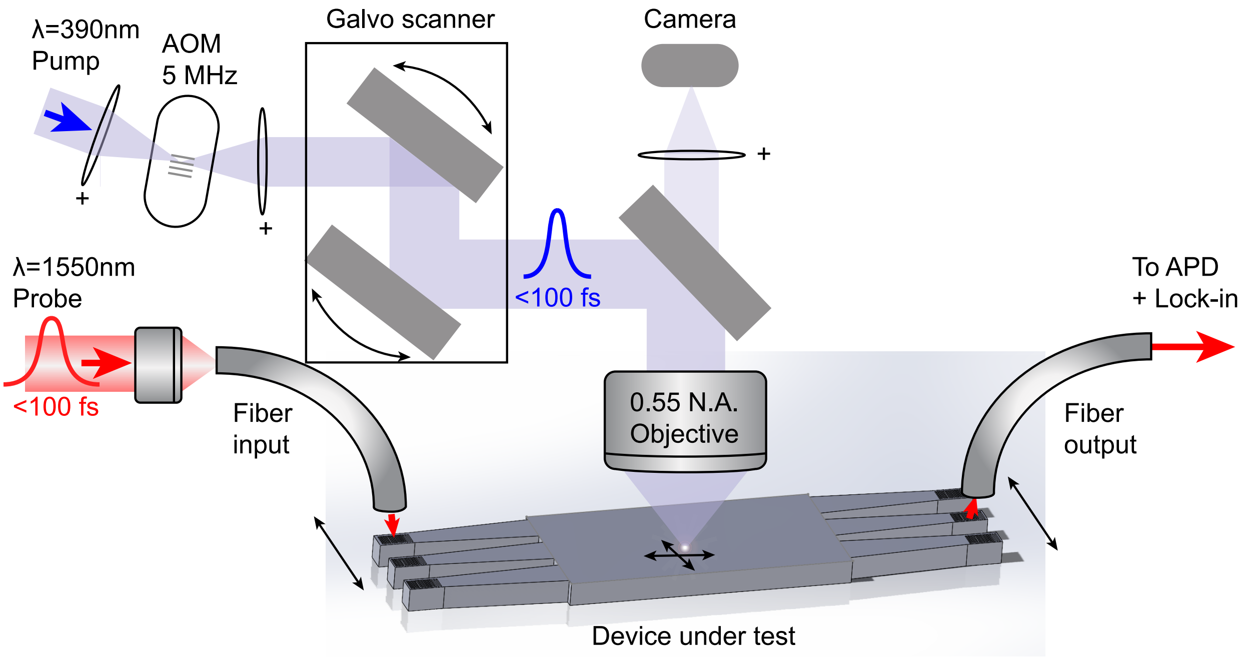

Figure S4: Schematic setup of UPMS technique, showing 1550 nm probe input coupled in and out of device using grating couplers.

4 Additional simulation results

4.1 Simulated device performance for 2×2 MMI

In addition to the near field maps presented in Figure 1 (c-d), we show here the electric field distribution of the other non-presented ports. To that end Figure SS5 demonstrates all port combinations for the patterned 2×2 MMI device for the bar (left) and cross (right) transformation.

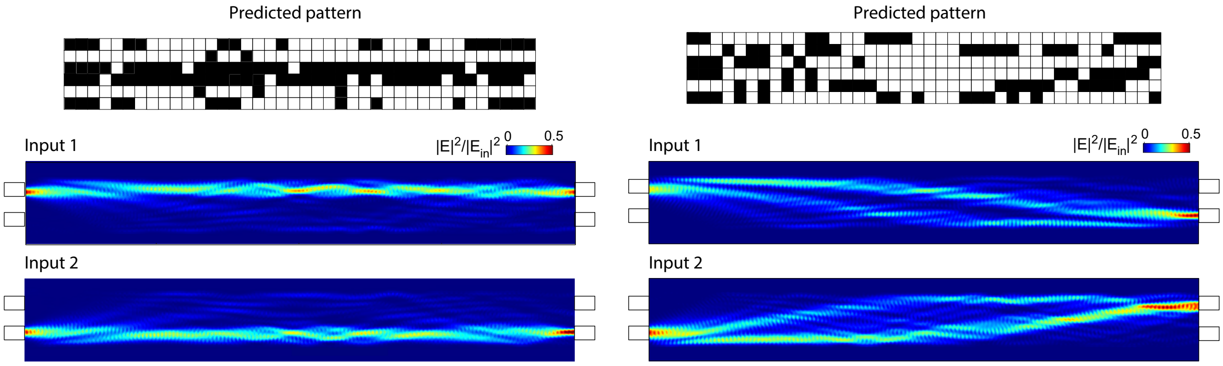

Figure S5: Simulated near field maps for all 4 ports of the patterned MMI device presented in Figure 1 of the main text.

4.2 Simulated broadband transmission for 4×4 MMI

Experimental validation of the broadband capability of programmed devices is shown in Figure 3 of the main text. In Figure S6 these results are validated by comparing the simulated device performance across all c-band wavelengths. The FDTD source wavelength is swept between 1530 nm and 1570 nm, allowing evaluation of the splitting ratio at a number of test wavelengths. The total device transmission (a) remains close to 70% regardless of the input port. (b) demonstrated the strong optical contrast between targeted ports, highlighted in the legend, and non-targeted outputs which remain below 10% transmission in all test cases.

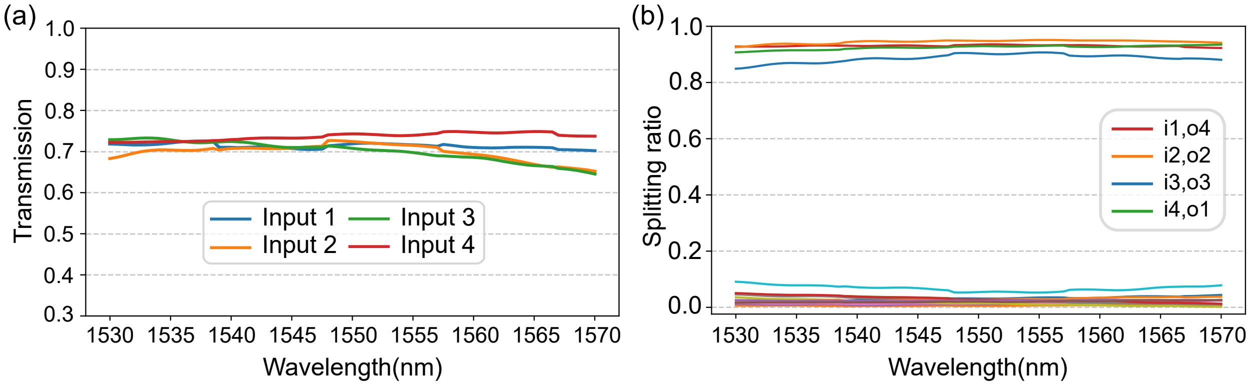

Figure S6: Simulated spectral dependence of a patterned 4×4 MMI as presented in Figure 4 of the main text. Across all C-band wavelengths the total device transmission remains around 70% (a) with a strong optical contrast shown in (b) between targeted (i1o4, i2o1, i3o3, i4o1) and non-targeted ports for the optimization.

4.3 Simulations of sensitivity to pattern misalignment and Sb_2Se_3 film thickness

In order to evaluate the sensitivity to experimental parameters we performed a number of simulations on a patterned 3×3 MMI, corresponding to Figure 2a of the main article. The pattern and geometry are illustrated in Figure S7a, with the simulated transmission matrix shown in Figure S7b for the ideal pattern alignment. Misalignments in the horizontal and vertical directions are defined as Δx and Δy respectively as illustrated in Figure S7a. Figure S7c shows the calculated port transmissions for the three main transmission channels corresponding to the largest matrix elements in Figure S7b, respectively $i_1 : o_3$, $i_2 : o_1$ and $i_3 : o_2$. it can be seen that the decrease in transmission T_{norm} is much stronger for the vertical (Δy) than for the horizontal (Δx) misalignment. Further analysis is presented in Figure S7d in terms of cosine similarity (blue dots) and excess insertion loss (red triangles). Generally, the insertion loss drops faster than the cosine similarity, indicating that overall device throughput is more sensitive to misalignment than the shape of the matrix itself. In the vertical direction, a misalignment of ±100 nm is seen to result in 1 dB additional loss, and 3 dB is reached at around ±200 nm misalignment. Cosine similarity stays relatively flat but drops steeply above $\Delta y = 200$ nm, illustrating an initial tolerance but rapid collapse of

the matrix structure itself as the pattern loses its alignment beyond this critical distance.

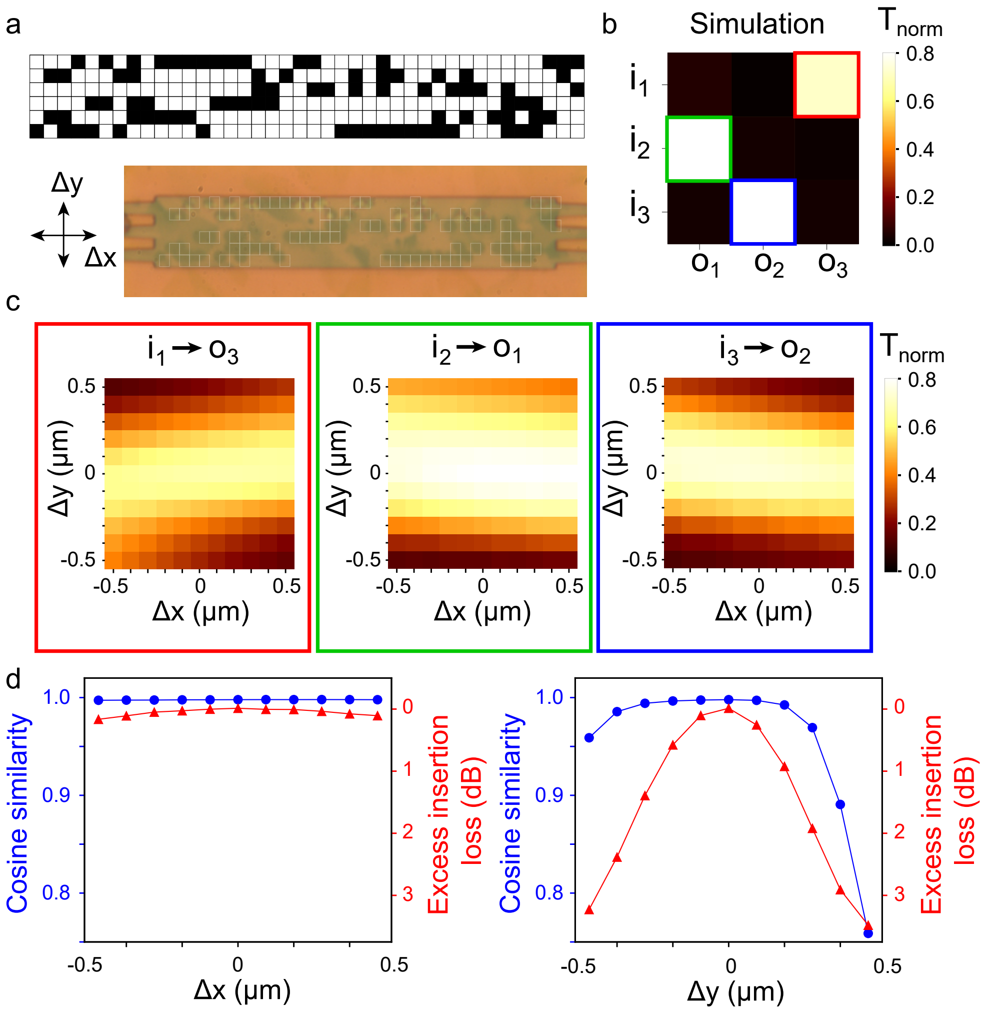

Figure S7: Simulation of effect of pixel pattern misalignment in X and Y directions on transmission matrix performance. (a) Pixel pattern with experimental map from main text Figure 2 used for this study. (b) Transmission matrix for simulated design with perfect alignment ($\Delta x = \Delta y = 0$). (c) Maps of three matrix elements (color coded according to (b)) against pattern misalignment in both Δx and Δy. (d) Cosine similarity and excess loss between matrices for optimized and misaligned case against misalignment for Δx and Δy separately.

In addition, simulations are carried out to illustrate the sensitivity of the device performance to the PCM thickness. As this is a parameter dependent on growth conditions and local variations, an accuracy of around ±5 nm could be expected under real world conditions. Variation of the Sb_2Se_3 thickness from 0 nm (no PCM) to 50 nm for a pattern optimized for

30 nm thickness shows a relatively flat region in which the shape of the matrix is preserved from around 20-40 nm. In this range, the excess insertion loss varies from 0.7 dB to -0.3 dB, where we observe that the slight reduction in our simulation indicates a small improvement in performance for slightly thicker PCM layer. Our model however does not include the increased polycrystalline scattering losses associated with thicker PCM films as seen experimentally.[2] Overall the simulation shows a fairly robust performance of the devices against typical variations in PCM thickness.

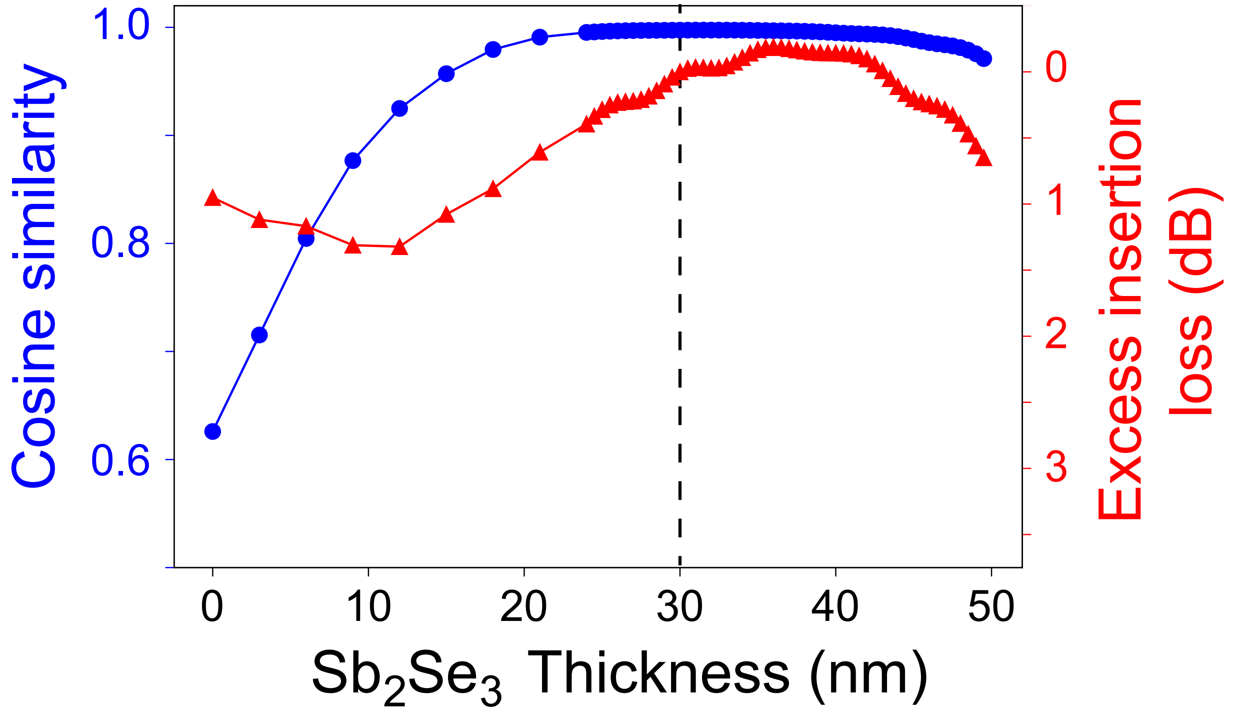

Figure S8: Effect of variation of Sb_2Se_3 thickness on transmission matrix performance. Cosine similarity and excess loss calculated for different Sb_2Se_3 thickness. Thickness of 30 nm was used as the design condition for the transmission matrix of Figure S7.

References

(1) Delaney, M.; Zeimpekis, I.; Du, H.; Yan, X.; Banakar, M.; Thomson, D. J.; Hewak, D. W.; Muskens, O. L. Nonvolatile programmable silicon photonics using an ultralow-loss Sb2Se3 phase change material. Science Advances **2021**, 7, eabg3500.

(2) Blundell, S.; Radford, T. W.; Ajia, I. A.; Lawson, D.; Yan, X.; Banakar, M.; Thomson, D. J.; Zeimpekis, I.; Muskens, O. L. Ultracompact programmable silicon photonics using layers of low-loss phase-change material Sb2Se3 of increasing thickness. ACS photonics **2025**, 12, 1382–1391.
